# Adipose-Derived Stem Cell Membrane-Coated Mitochondria Restore Tendon Stromal Cell Function Through Metabolic Reprogramming and Promote Achilles Tendon Healing

**DOI:** 10.3390/jfb17030119

**Published:** 2026-03-02

**Authors:** Xu Li, Ziqi Huo, Zeyu Wang, Haoyuan Deng, Hongwei Shao, Ye Li, Chunyan Jiang

**Affiliations:** 1 Department of Sports Medicine, Beijing Jishuitan Hospital, Capital Medical University, Beijing 100035, China; 2 Musculoskeletal Research Laboratory, Department of Orthopaedics & Traumatology, Faculty of Medicine, The Chinese University of Hong Kong, Hong Kong 999077, China; 3 Department of Rehabilitation Sciences, The Hong Kong Polytechnic University, Hong Kong 999077, China

**Keywords:** tendon repair, mitochondrial delivery, metabolic modulation, membrane-coated nanoparticles, oxidative phosphorylation

## Abstract

Achilles tendon rupture often leads to poor functional recovery due to limited self-healing, with mitochondrial dysfunction in tendon stromal cells (TSCs) being a key factor in disease progression. Here, we developed adipose-derived stromal cell (ADSC) membrane-coated mitochondria (Mito-NPs) to target this dysfunction and evaluate their therapeutic potential for tendon repair. Mito-NPs exhibited uniform size, stable surface charge, and effective membrane coating. In lipopolysaccharide-induced inflammatory TSCs, Mito-NPs enhanced oxidative phosphorylation, improved mitochondrial metabolic homeostasis, and reshaped gene expression profiles to normalize TSC functional phenotypes, including inflammation, migration, and collagen synthesis. When encapsulated in a reactive oxygen species (ROS)-responsive hydrogel (Mito-NPs@HG) and implanted into rat Achilles tendon injuries, Mito-NPs@HG improved gait function, decreased local inflammation, and promoted histological repair of damaged tendons by enhancing collagen organization and reducing inflammation. Our findings demonstrate that ADSC membrane-coated mitochondria effectively rescue TSC dysfunction and facilitate tendon regeneration, providing a promising translational strategy for treating tendon injuries.

## 1. Introduction

Achilles tendon rupture is a prevalent sports-related injury, with global incidence rising due to increased participation in athletic activities [1,2]. The annual incidence of Achilles tendon rupture ranges from 5 to 50 cases per 100,000 individuals [3,4,5]. Inadequate treatment of Achilles tendon rupture can result in substantial loss of heel-lifting strength and abnormal gait, which negatively impact daily activities and occupational performance [6]. Current interventions, such as direct suturing, autografts, and allografts, are limited by factors including insufficient mechanical strength, risk of immune rejection, donor site complications, and poor long-term functional outcomes [7]. These complications often manifest as tendon retear, peritendinous adhesion, and restricted range of motion [7].

One key factor contributing to the failure of tendon regeneration is a pathological microenvironment characterized by high levels of reactive oxygen species (ROS), which trigger acute oxidative stress and lead to DNA damage, collagen degradation, tenocyte apoptosis, and M1 macrophage polarization [8,9]. Mitochondria serve as the primary energy source within cells and are also the main site of ROS generation [10]. Under physiological conditions, the mitochondrial electron transport chain produces minimal ROS during aerobic respiration [10]. However, mitochondrial dysfunction caused by injury or stress leads to electron leakage and excessive ROS production, which can induce oxidative damage to cellular components, including lipids, proteins, and nucleic acids. This process can trigger a vicious cycle where ROS overproduction worsens mitochondrial impairment, which then increases ROS production [11,12]. ROS, a byproduct of oxidative phosphorylation (OXPHOS), also acts as a key signaling molecule modulating tenocyte activity [13]. Clinical studies have indicated that elevated superoxide-induced oxidative stress is associated with recurrent tendon tears following rotator cuff repair [13,14]. In tendon stromal cells, the scavenging of ROS can regulate the differentiation, inhibit tendon calcification, and promote collagen formation [15]. As central regulators of energy metabolism [16], mitochondria are involved in various biological processes that maintain tendon homeostasis [9]. Mitochondrial dysfunction contributes to tendinopathy through processes such as inflammation, apoptosis, and adipocyte infiltration of tenocytes [17]. Degenerated tendons exhibit reduced ATP synthesis and downregulated expression of electron transport chain-related genes (e.g., ATP5F1A, Fxn, oPA1) [18]. These abnormalities in mitochondrial structure and function were correlated with diminished tendon biomechanical strength [18].

Recently, mitochondrial transfer has emerged as a promising therapeutic strategy for enhancing tissue repair [9,19,20,21]. This technique involves isolating functional mitochondria from healthy tissues and delivering them to injured sites via local or systemic injection. The underlying mechanisms include restoring energy metabolism, attenuating inflammatory responses, and inhibiting apoptosis [20,22]. Among the donor sources for mitochondrial transfer, stem cells serve as the primary carriers for endogenous mitochondrial transfer [22]. Transplantation of intact mitochondria from L6 cells has been confirmed to exert a protective effect against TNFα-induced tendinopathy [23]. Tendon repair requires more accessible methods for mitochondrial delivery and rigorous validation of the efficacy of mitochondrial transplantation. From a clinical translation perspective, adipose-derived stromal cells (ADSCs) hold great promise as ideal seed cells for exogenous mitochondrial transplantation, owing to their unique advantages [22]. ADSCs can be conveniently isolated via minimally invasive liposuction, which causes minimal damage to the donor [24,25]. They exhibit robust proliferative capacity and maintain viability easily under in vitro culture conditions [25,26]. This not only lays a foundation for the subsequent large-scale preparation of autologous mitochondria but also effectively reduces the risk of immune rejection that may arise from allogeneic transplantation [21].

In this study, we developed a ROS-responsive hydrogel incorporating mitochondria coated with ADSC membranes to promote the functional recovery of injured tendons. ADSC membranes and mitochondria were first isolated and combined by extrusion to form Mito-NPs, whose physicochemical properties were thoroughly characterized. We then assessed the impact of Mito-NPs on tendon stromal cell (TSC) function under lipopolysaccharide (LPS)-induced inflammatory conditions, focusing on proliferation, migration, collagen synthesis, and mitochondrial metabolism. Transcriptomic analysis provided insight into the molecular mechanisms underlying these effects. Finally, the therapeutic efficacy of Mito-NPs encapsulated within the ROS-responsive hydrogel (Mito-NPs@HG) was validated in vivo using a rat Achilles tendon injury model. Collectively, this work introduces a cell membrane-coated mitochondrial delivery platform with significant potential to enhance tendon repair and functional recovery.

## 2. Materials and Methods

### 2.1. Primary ADSCs Isolation and Culture

Four-week-old male Sprague-Dawley (SD) rats were euthanized using isoflurane inhalation anesthesia. Inguinal fat pads were excised after disinfecting with 75% ethanol for 10 min. The adipose tissue was minced into 1 to 2 mm^3^ pieces with sterile fine scissors and digested in 0.1% type I collagenase (Sigma-Aldrich, St. Louis, MO, USA) at 37 °C for 1 h on a shaker set at 150 rpm. Digestion was stopped by adding Dulbecco’s Modified Eagle Medium (DMEM, Gibco, Waltham, MA, USA) with 10% fetal bovine serum (FBS, Gibco, Waltham, MA, USA). The cell suspension was filtered through a 100-mesh sterile sieve to remove undigested tissue debris and centrifuged at 1500× *g* rpm at 4 °C for 5 min. The supernatant was discarded, and the cell pellet was resuspended in DMEM containing 10% FBS and 1% penicillin-streptomycin (P/S, Gibco, Waltham, MA, USA). Cells were seeded in 100 mm culture dishes and incubated at 37 °C in a 5% CO_2_ atmosphere. The culture medium was changed every 2 days. Cells were used for subsequent experiments once they reached approximately 90% confluence.

### 2.2. Primary TSCs Isolation and Culture

Four-week-old male SD rats were euthanized under anesthesia and disinfected with 75% ethanol. Achilles tendons were excised, cleared of surrounding connective tissue, and minced into 1 mm^3^ fragments. The tissue fragments were plated in 6-well plates and cultured in Dulbecco’s Modified Eagle Medium (DMEM, Gibco, Waltham, MA, USA) supplemented with 10% FBS and 1% P/S. Plates were incubated at 37 °C in a 5% CO_2_ atmosphere, and the medium was replaced every 3 days. When TSCs migrated from the tissue fragments and reached 90% confluence, they were passaged using 0.25% trypsin-EDTA (Gibco, Waltham, MA, USA) and utilized for experiments at passages 3 to 5.

### 2.3. Cell Membrane Extraction

Cell membranes were isolated from ADSCs using a modified protocol. ADSCs at 90% confluence were harvested with trypsin-EDTA, washed twice with cold phosphate-buffered saline (PBS), and resuspended in IB-1 buffer at pH 7.4. The buffer contained 0.5% bovine serum albumin (BSA), 225 mM mannitol, 30 mM Tris-HCl, 0.5 mM EDTA, 75 mM sucrose, 20 µL protease inhibitor per 4 mL buffer, and 1% phosphatase inhibitor. The cell suspension was sonicated on ice at 300 W for 10 cycles of 5 s on and 10 s off using a probe sonicator. The mixture was centrifuged at 800× *g* and 4 °C for 10 min to remove intact cells and nuclei. The supernatant was collected and centrifuged at 10,000× *g* and 4 °C for 20 min. The resulting pellet was ultracentrifuged at 100,000× *g* and 4 °C for 60 min. The final membrane pellet was lyophilized for 24 h, weighed, and stored at −80 °C until use.

### 2.4. Mitochondrial Extraction and Cell Membrane-Mitochondria Encapsulation

Mitochondria were isolated from ADSCs using the Mitochondria Isolation Kit for Cultured Cells (Beyotime Biotechnology, Shanghai, China) following the manufacturer’s instructions. The protein concentration of the isolated mitochondria was measured with the bicinchoninic acid (BCA) Protein Assay Kit (Beyotime Biotechnology, Shanghai, China).

For encapsulation, the lyophilized cell membrane was resuspended in ultrapure water to a concentration of 2 mg/mL. An equal volume of mitochondrial suspension, also at 2 mg/mL as determined by the BCA assay, was added. The combined suspension was incubated at 37 °C for 30 min. The mixture was then extruded sequentially through polycarbonate membranes with pore sizes of 1000 nm, 400 nm, and 200 nm using a mini-extruder at room temperature. The resulting cell membrane-encapsulated mitochondria, referred to as Mito-NPs, were collected by centrifugation at 10,000× *g* and 4 °C for 5 min and resuspended in PBS.

For transmission electron microscope (TEM) analysis, Mito-NPs were fixed, loaded onto carbon-coated copper grids, and negatively stained with uranyl acetate. After air-drying, samples were imaged under a TEM to characterize morphology and structural features.

### 2.5. Fluorescence Colocalization Detection of Mito-NPs

ADSC membranes were labeled with 1 μM Dil (Thermo Fisher Scientific, Waltham, MA, USA) at 37 °C for 30 min. Isolated mitochondria were labeled with 200 nM Mito-Tracker Green (Thermo Fisher Scientific, Waltham, MA, USA) at 37 °C for 20 min. Free dye was removed by centrifugation (100,000× *g* for 60 min for membranes; 10,000× *g* for 10 min for mitochondria) and buffer exchange with PBS. Labeled membranes and mitochondria were combined at a 1:1 volume ratio and extruded sequentially through 1000 nm, 400 nm, and 200 nm polycarbonate membranes. The resulting Mito-NPs were immediately deposited onto glass coverslips, mounted in anti-fade medium (Thermo Fisher Scientific, Waltham, MA, USA), and imaged using a laser-scanning confocal microscope (Zeiss LSM 900, Oberkochen, Germany). Dil and Mito-Tracker Green fluorescence signals were acquired, and colocalization was quantified using Zeiss ZEN 3.4 software.

### 2.6. Detection of Mitochondrial Membrane Potential Before and After Extrusion

Mitochondrial membrane potential was assessed using the JC-1 fluorescent probe (Beyotime, Shanghai, China, C2006). Briefly, both unextruded mitochondria and extruded mitochondria (after ADSC membrane encapsulation) were incubated with JC-1 staining solution at 37 °C for 20 min in the dark. After incubation, the samples were washed twice with JC-1 buffer to remove unbound dye. The fluorescence intensity of JC-1 monomers (green fluorescence, FITC channel) and J-aggregates (red fluorescence, PE channel) was detected by flow cytometry. The ratio of green to red fluorescence was calculated to evaluate changes in mitochondrial membrane potential.

### 2.7. Hydrogel Synthesis and CM-Mito-NPs Encapsulation

A ROS-responsive hydrogel was synthesized as previously described using polyvinyl alcohol (PVA) and 4-sulfophenylboronic acid (TSPBA), which form reversible boronate ester bonds sensitive to ROS [27]. The synthesis utilized PVA (Sigma-Aldrich, St. Louis, MO, USA), TSPBA (Aladdin, Shanghai, China), and ultrapure water (Millipore, Burlington, MA, USA). For PVA solution preparation, 4.5 g PVA was dissolved in 100 mL ultrapure water by heating to 95 °C with magnetic stirring at 300 rpm for 15 min. The solution was cooled to 25 °C to yield an 4.5% (*w*/*v*) PVA stock, then filtered through a 0.22 μm sterile filter (Millipore, Burlington, MA, USA). For TSPBA cross-linker preparation, 0.45 g TSPBA was dissolved in 10 mL ultrapure water to obtain a 4.5% (*w*/*v*) TSPBA stock. To form the hydrogel and encapsulate Mito-NPs, the sterile 4.5% PVA solution was added with Mito-NPs and mixed with 4.5% TSPBA at a 1:1 volume ratio. The mixture was incubated at 25 °C for 10 min to complete cross-linking.

### 2.8. ROS Responsiveness of Hydrogels

Hydrogel responsiveness to ROS was assessed by measuring changes in wet weight after exposure to hydrogen peroxide (H_2_O_2_). Hydrogels were incubated in 1 mL PBS with 0 or 100 μM H_2_O_2_ at 37 °C. At specified time points (0, 2, and 4 h), hydrogels were removed, blotted to remove surface moisture, and weighed using an electronic analytical balance. Wet weight retention was calculated as (W_t_/W_0_) × 100%, where W_t_ indicates the wet weight at each time point and W_0_ is the initial wet weight.

### 2.9. Hydrogel Degradation and Mito-NP Release

Hydrogel degradation assays were conducted by preparing 100 μL of hydrogel and incubating it in 1 mL of PBS at 37 °C. At 24, 48, and 72 h, the supernatant was discarded, and the wet weight of the hydrogel was measured to determine the percentage of mass loss.

For Mito-NP release experiments, Mito-NPs were labeled with Dil before hydrogel encapsulation. The hydrogel-encapsulated Dil-labeled Mito-NPs were incubated in 1 mL of PBS at 37 °C. At 24, 48, and 72 h post-incubation, aliquots of the supernatant were collected to quantify the fluorescence intensity of Dil. As a control, free Dil-labeled Mito-NPs (without hydrogel encapsulation) were incubated in 1 mL of PBS under identical conditions, and the total fluorescence intensity of this group was used to represent the total amount of Mito-NPs.

### 2.10. TSC Treatment

TSCs were seeded in 96-well plates at a density of 2 × 10^3^ cells per well, treated with ADSC membranes and Mito-NPs at various concentrations, and subjected to cell viability analysis using the CCK-8 (Beyotime, Shanghai, China, C0038) assay at 24 h post-treatment.

TSCs were seeded into 24-well plates (2 × 10^4^ cells per well) or confocal dishes (5 × 10^4^ cells per dish). The normal group was cultured in standard DMEM. The LPS group was treated with 1 μg/mL LPS, the LPS + Mito-NPs group received co-treatment with 1 μg/mL LPS and 5 μg/mL Mito-NPs, and the LPS +cell membrane group received co-treatment with 1 μg/mL LPS and 5 μg/mL ADSC membrane. All groups were incubated at 37 °C in a 5% CO_2_ humidified atmosphere for 24 h before subsequent analyses.

### 2.11. Mitochondrial Content Detection

TSCs cultured in confocal dishes were stained with 100 nM Mito-Tracker Red CMXRos (Beyotime Biotechnology, Shanghai, China, C1035) at 37 °C for 20 min. Following PBS washes, nuclei were counterstained with 1× Hoechst 33342 (Beyotime Biotechnology, Shanghai, China) for 5 min. Images were acquired using a Zeiss LSM 900 confocal microscope.

### 2.12. Intracellular ROS Detection 

Intracellular ROS levels were determined using the DCFH-DA ROS Detection Kit (Beyotime, Shanghai, China). TSCs were incubated with 1× DCFH-DA at 37 °C for 30 min, washed with PBS, and stained with 1× Hoechst 33342 for 5 min. Images were acquired using a Zeiss LSM 900 confocal microscope.

### 2.13. Glucose and Fatty Acid Uptake Detection

Glucose uptake was assessed by incubating TSCs with 300 μM 2-NBDG (Thermo Fisher Scientific, Waltham, MA, USA) for 24 h as previously described [28]. Fatty acid uptake was measured by incubating TDSCs with 1 μM BODIPY 558/568 C12 (Red-C12, Thermo Fisher Scientific, Waltham, MA, USA) for 24 h as previously described [28]. Following incubation, cells were washed with PBS and stained with 1× Hoechst 33342. Images were acquired using a Zeiss LSM 900 confocal microscope.

### 2.14. Immunofluorescence Staining

TSCs cultured in 24-well plates were fixed with 70% ethanol at 4 °C for 30 min, washed three times with PBS, and permeabilized with 0.1% Triton X-100 (Sigma-Aldrich, St. Louis, MO, USA) for 15 min. After blocking with 1% BSA in PBST at room temperature for 1 h, cells were incubated overnight at 4 °C with primary antibodies against Collagen type I (Col1, Cat. No.: A24112), Collagen type III (Col3, Cat. No.: A3795), matrix metalloproteinase 9 (MMP9, Cat. No.: A25299), matrix metalloproteinase 13 (MMP13, Cat. No.: A11755), interleukin-6 (IL-6, Cat. No.: A0286), inducible nitric oxide synthase (iNOS, Cat. No.: A25899), TFAM (Cat. No.: A13552), Ki67 (Cat. No.: A20018), and tumor necrosis factor-alpha (TNF-α, Cat. No.: A21265) (all Abclonal, 1:400 dilution). After three PBS washes, cells were incubated with Alexa Fluor Cy3-conjugated secondary antibody (Thermo Fisher Scientific, Waltham, MA, USA, 1:100) at room temperature for 1 h. And then cells were stained with DAPI, and Actin-Tracker Green (C1033, Beyotime Biotechnology, Shanghai, China) for 30 min. Confocal images were acquired using a Zeiss LSM 900 microscope.

### 2.15. Seahorse Mitochondrial OXPHOS Analysis

TSCs were seeded in XF24 cell culture microplates (Agilent Technologies, Santa Clara, CA, USA) at 2 × 10^4^ cells per well and assigned to three groups (Ctrl, LPS, LPS + Mito-NPs). After 24 h of treatment, the culture medium was replaced with XF Base Medium (Agilent Technologies, Santa Clara, CA, USA) containing 10 mM glucose, 2 mM glutamine, and 1 mM pyruvate. Cells were incubated at 37 °C without CO_2_ for 1 h. Mitochondrial respiration was assessed using the Seahorse XF Cell Mito Stress Test Kit (Agilent Technologies, Santa Clara, CA, USA) on a Seahorse SF24 Extracellular Flux Analyzer, following the manufacturer’s protocol. Sequential injections of 1 μM oligomycin, 1 μM FCCP, and 0.5 μM rotenone/antimycin A were performed. Oxygen consumption rate (OCR) and parameters such as basal respiration, ATP production, maximal respiration, and spare respiratory capacity were calculated using Wave Desktop and Controller 2.6 Software (Agilent Technologies, Santa Clara, CA, USA).

### 2.16. Rat Achilles Tendon Rupture Model Construction

All animal procedures received approval from the Animal Ethics Committee of Beijing Jishuitan Hospital (AEEC No:2024-0805, 2024-0806). Eight-week-old male SD rats were randomly assigned to three groups: Sham control group (Achilles tendon exposure without rupture), Vehicle control group (Achilles tendon rupture treated with primary suture and implantation of vehicle), and NPs@HG Implant group (Achilles tendon rupture treated with primary suture and implantation of Mito-NPs@HG). The mitochondrial injection dose was standardized at 40 μg per rat, based on the previously reported dose range (~20 μg) [29,30]. Our preliminary experiments indicated that this dose of 40 μg provided optimal histological repair in this study.

Anesthesia was induced and the right hindlimb was disinfected with 75% ethanol, and a 1 cm longitudinal incision was made over the Achilles tendon. In the Suture and Implant groups, the Achilles tendon was transected at the midpoint with a scalpel. The Suture control group underwent end-to-end repair with 5-0 nylon thread, while the other two groups received suturing followed by hydrogel implantation at the suture site. Incisions were closed with 5-0 absorbable suture. Rats were euthanized at 1 and 4 weeks post-surgery, and Achilles tendon tissues were collected for analysis.

Rat Gait analysis was performed 4 weeks post-surgery using the Gait analysis system (ZS-BT/S, Beijing Zhongshi Dichuang Technology Development Co., Ltd., Beijing, China). Rats were acclimated to the test chamber for 30 min before measurement. During testing, rats walked freely along the glass walkway, and valid runs were recorded. Gait parameters were analyzed using the company’s software.

### 2.17. Tissue Processing and Histological Analysis

Harvested Achilles tendon tissues were fixed in 4% paraformaldehyde at 4 °C for 24 h, then decalcified in 0.5 M EDTA (pH 7.2–7.4) for 2 weeks with solution changes every three days. Tissues were dehydrated through a graded ethanol series, cleared in xylene, and embedded in paraffin. Serial 5 μm sections were prepared using a microtome (Leica RM2255, Nussloch, Germany) and mounted on glass slides. Hematoxylin and Eosin (H&E) staining (BKMAMLAB, Changsha, China), Masson’s trichrome (Solarbio Life Science, Beijing, China) and Sirius Red (Solarbio Life Science, Beijing, China) staining were conducted following the manufacturer’s guidelines. Stained sections were imaged using a brightfield microscope (Olympus BX53, Hachioji, Japan). Immunohistochemistry

Paraffin sections were dewaxed in water, treated with 3% H_2_O_2_ (methanol solution) at room temperature for 10 min to quench endogenous peroxidase activity, and subjected to antigen retrieval in 0.01 M citrate buffer (pH 6.0) at 95 °C for 20 min. Sections were permeabilized with 0.3% Triton X-100 for 15 min, blocked with 5% normal goat serum (Solarbio, Beijing, China) at room temperature for 1 h, and incubated with primary antibodies against Col1 (1:200), Col3 (1:200), MMP9 (1:200), MMP13 (1:200), IL-6 (1:200), and TNF-α (1:200) at 4 °C overnight. After PBS washes, sections were incubated with HRP-conjugated secondary antibody (Abclonal, Woburn, MA, USA, 1:500) at room temperature for 1 h. Signals were developed with 3,3′-diaminobenzidine (DAB, Thermo Fisher Scientific, Waltham, MA, USA) substrate, and nuclei were counterstained with hematoxylin. Sections were dehydrated, cleared, and mounted. Images were captured under a light microscope.

### 2.18. RNA Sequencing

For in vitro experiments, TDSCs from the three groups (Ctrl, LPS, LPS + Mito-NPs) were collected after 24 h of treatment, washed twice with PBS, and snap-frozen in liquid nitrogen (*n* = 3 per group). For in vivo experiments, Achilles tendon tissues from the three groups (Sham, Suture, Implant) were harvested at four weeks post-surgery, rinsed with PBS to remove blood, and snap-frozen in liquid nitrogen (*n* = 3 per group).

Total RNA was extracted from samples using TRIzol Reagent (Thermo Fisher Scientific, Waltham, MA, USA) according to the manufacturer’s instructions. Library construction and sequencing were performed on the Illumina NovaSeq 6000 platform by Novogene Co., Ltd. (Beijing, China). Raw sequencing data were filtered to remove low-quality reads and adapter sequences. Clean reads were aligned to the rat reference genome (Rnor_6.0) using HISAT2. Gene expression levels were quantified as fragments per kilobase of transcript per million mapped reads (FPKM) using StringTie. Differentially expressed genes (DEGs) were identified using DESeq2 with |log_2_(fold change)| > 1 and adjusted *p* < 0.05. Gene Ontology (GO) and Kyoto Encyclopedia of Genes and Genomes (KEGG) enrichment analyses of DEGs were performed using clusterProfiler. Raw sequencing data were deposited in the NCBI SRA (SUB15730792 and SUB15730881).

### 2.19. Statistical Analysis

All data were analyzed using GraphPad Prism 10.0 software. Quantitative results are presented as mean ± standard deviation (SD). Comparisons between two groups were performed using a one-tailed unpaired *t*-test. Comparisons among three or more groups were conducted using one-way analysis of variance (ANOVA) followed by Tukey’s post hoc test or two-way ANOVA by Fisher’s LSD test. Statistical significance was defined as *p* < 0.05, with significance levels indicated as * *p* < 0.05, ** *p* < 0.01, *** *p* < 0.001, and **** *p* < 0.0001. Sample sizes (*n*) are specified in each experiment’s description.

## 3. Results

### 3.1. Preparation, Characterization, and Cellular Uptake of Mito-NPs

**Figure 1:**
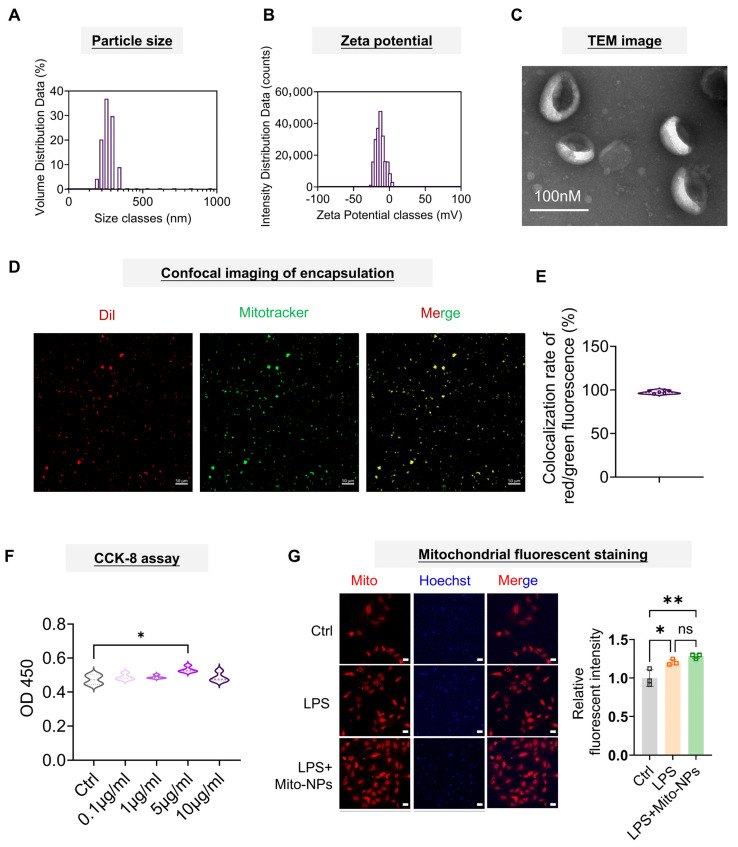
Characterization of Mito-NPs. (**A**). Nanoparticle size distribution plot of Mito-NPs. (**B**). Zeta potential plot of Mito-NPs. (**C**). Representative TEM image of Mito-NPs. Scale bar indicates 100 nm. (**D**). Representative confocal microscope image of Mito-NPs. Dil labels the membranes of ADSC, and Mitotracker Green labels mitochondria (green). Scale bar indicates 50 μm. (**E**). Quantification of the colocalization of Dil and Mitotracker signals. *n* = 4. (**F**). CCK8 assay for detection of the effect of Mito-NPs on the proliferation of TSCs was detected by the CCK-8 assay. *n* = 4. (**G**). Mitochondria staining of TSC in control, LPS, and LPS + Mito-NPs groups and the quantification analysis. The nucleus was labeled with Hoechst (Blue). Mitotracker labels mitochondria (red). *n* = 3. Scale bar indicates 50 μm. * *p* < 0.05, ** *p* < 0.01, *** *p* < 0.001; All data are shown as the mean ± SD; Statistical significance was determined by one-way ANOVA with Turkey’s test.

Membranes and mitochondria were isolated from rat ADSCs, and Mito-NPs were produced using the extrusion method. DLS analysis showed that Mito-NPs had a uniform particle size distribution, with an average diameter of 265.03 nm and a Zeta potential of −12.32 mV (Figure 1A,B), aligning with the expected negative surface charge of the adipose stem cell membrane [31]. TEM displayed a vesicle-like structure for the Mito-NPs (Figure 1C). Fluorescence co-localization experiments revealed a 97.3% overlap between Mitotracker Green-labeled mitochondria and Dil-labeled ADSC membranes, confirming the coating of the mitochondria by the ADSC membrane (Figure 1D,E). Flow cytometry analysis of JC-1 staining revealed that the ratio of green to red fluorescence did not increase but rather decreased significantly after mitochondrial encapsulation via extrusion, indicating that the extrusion process did not impair mitochondrial membrane potential (Supplementary Figure S1).

TSCs were exposed to Mito-NPs at concentrations ranging from 0.1 to 10 μg/mL (0.1, 1, 5, and 10 μg/mL) (Figure 1F). The CCK-8 assay revealed that treatment with 5 μg/mL Mito-NPs for 24 h resulted in a significantly higher proliferation rate than the control. However, the ADSC membrane at the same concentration range did not exhibit a similar pro-proliferative effect (Supplementary Figure S2). The concentration of 5 μg/mL was determined to be optimal and used in subsequent experiments (Figure 1F). TSCs were exposed to 1 μg/mL LPS for 24 h to establish an inflammatory microenvironment. The mitochondrial fluorescence intensity in the LPS group was significantly higher than in the control group. After treatment with Mito-NPs, mitochondrial fluorescence intensity in TSCs increased even more (Figure 1G).

### 3.2. Mito-NPs Enhance Mitochondrial Metabolism of TSCs Within an Inflammatory Microenvironment

**Figure 2:**
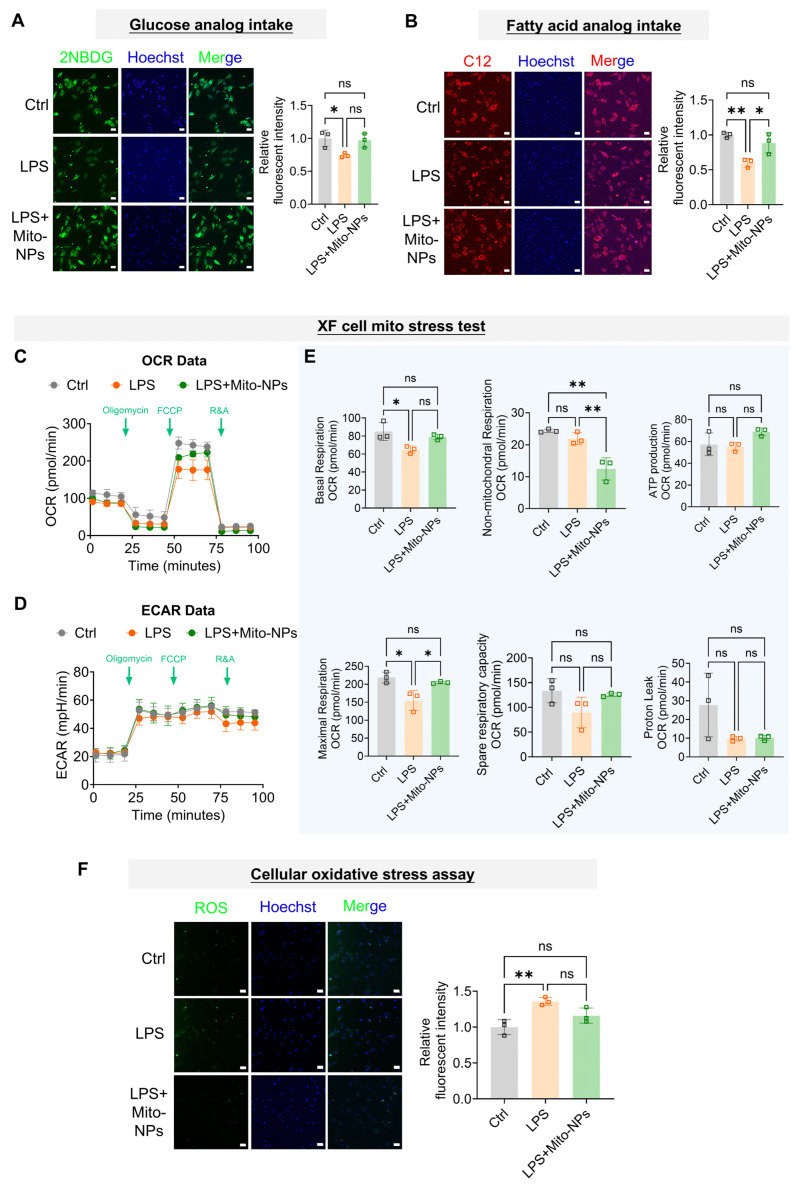
The effect of Mito-NPs on the mitochondrial metabolism of TSCs under inflammatory conditions in vitro. (**A**). Representative confocal images showing 2NBDG (Green) intensity in TSCs under control, LPS, and LPS + Mito-NPs conditions and quantification analysis. The nucleus was labeled with Hoechst (Blue). Scale bar indicates 50 μm. (**B**). Representative confocal images showing C12 (Red) intensity in TSC under control, LPS, and LPS + Mito-NPs conditions and quantification analysis. The nucleus was labeled with Hoechst (Blue). Scale bar indicates 50 μm. (**C**). Real-time recording of the OCR of TSCs under control, LPS, and LPS + Mito-NPs conditions. (**D**). Real-time recording of the ECAR of TSCs under control, LPS, and LPS + Mito-NPs conditions. (**E**). Quantitative analysis of key mitochondrial respiratory parameters, including basal respiration, ATP production, proton leak, maximal respiration, spare respiratory capacity, and non-mitochondrial respiration. (**F**). Representative confocal images showing ROS levels in TSCs under control, LPS, and LPS + Mito-NPs conditions and quantification analysis. The nucleus was labeled with Hoechst (Blue). Scale bar indicates 50 μm. *n* = 3. * *p* < 0.05, ** *p* < 0.01, *** *p* < 0.001; All data are shown as the mean ± SD; Statistical significance was determined by one-way ANOVA with Turkey’s test.

Inflammatory responses were known to impair cellular energy metabolism, such as glucose and fatty acid uptake [32]. To investigate whether mitochondrial delivery could rescue this suppression, we assessed the uptake of fluorescently labeled metabolic analogs—2-NBDG (glucose) and C12 (fatty acid)—in TSCs. LPS treatment significantly reduced the uptake of both substrates. However, supplementation with 5 μg/mL Mito-NPs in the LPS-induced inflammatory environment markedly restored their uptake (Figure 2A,B). Consistent with these findings, Seahorse analysis revealed that LPS concurrently suppressed both oxidative phosphorylation (OCR) and glycolysis (ECAR) (Figure 2C–E). Mito-NPs partially reversed this dysfunction, indicating a protective effect on mitochondrial function in TSCs under inflammatory conditions (Figure 2C–E). The lower basal and maximum respiration rates in the LPS group further confirmed the mitochondrial impairment (Figure 2C,E). This suggested that the increased mitochondrial fluorescence intensity induced by LPS (Figure 1G) likely reflected a compensatory increase mitochondrial biogenesis in response to inflammation. Interestingly, treatment with Mito-NPs significantly upregulated the expression of mitochondrial transcription factor A (TFAM) under LPS-induced inflammatory conditions, confirming the activation of endogenous mtDNA replication (Supplementary Figure S3). In contrast, ADSC membrane failed to exert a comparable effect. Furthermore, ROS detection with DCFH-DA showed a significant increase in ROS levels in the LPS group, which was mitigated by Mito-NPs treatment (Figure 2F).

### 3.3. Transcriptional Profile of TSCs Regulated by Mito-NPs

**Figure 3:**
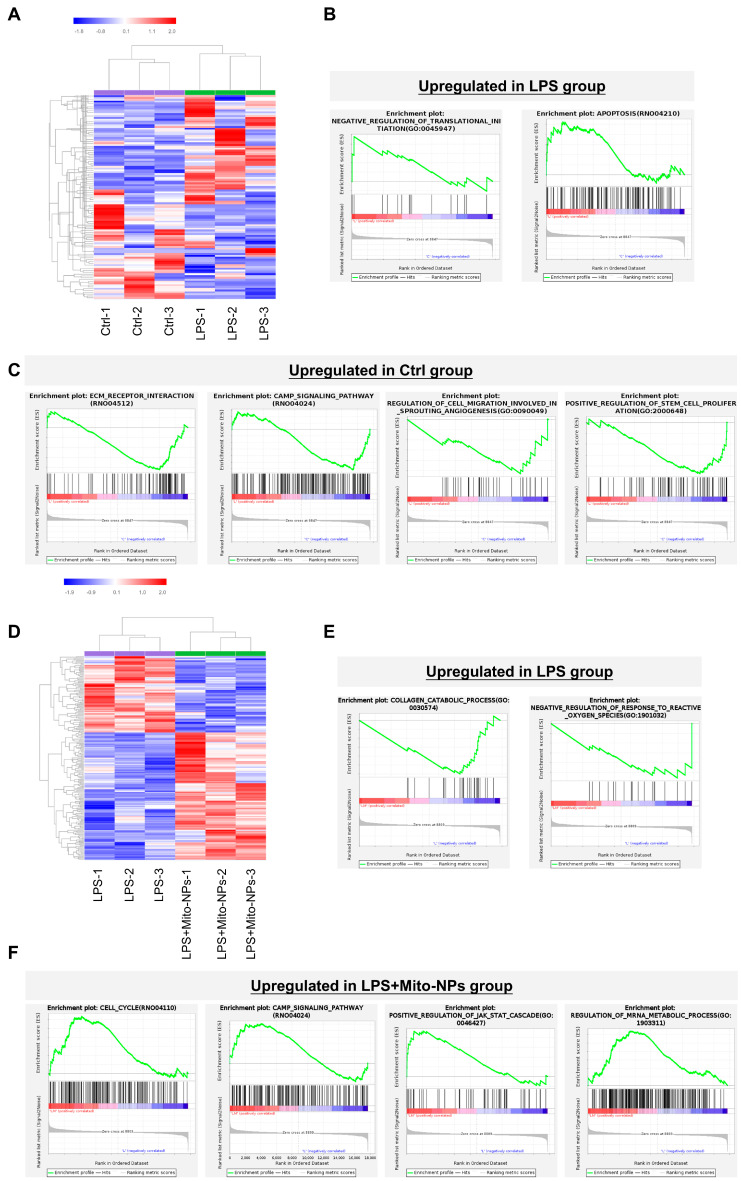
Transcriptome analysis of TSCs in vitro. (**A**,**B**) Differential gene enrichment analysis of the control group and LPS group. (**A**). Heatmap of differentiated expression genes between the Control and LPS groups. (fold change, ≥1; *p* value < 0.05) (**B**). GSEA plots of the gene sets that is upregulated in the LPS group. (**C**). GSEA plots of the gene sets that is upregulated in the control group. (**D**). Heat map of differentiated expression genes between LPS and LPS + Mito-NPs groups. (fold change, ≥1; *p* value < 0.05) (**E**,**F**) Differential gene enrichment analysis of the LPS group and LPS + Mito-NPs. (**E**). GSEA plots of the gene sets that is upregulated in the LPS group. (**F**). GSEA plots of the gene sets that is upregulated in the LPS + Mito-NPs group. *n* = 3.

Transcriptome sequencing was performed to examine functional alterations in TSCs under LPS-induced inflammatory conditions and following Mito-NPs treatment. Differential gene analysis and gene set enrichment analysis (GSEA) showed that genes up-regulated in the LPS group were mainly linked to negative regulation of translational initiation and apoptosis-related pathways (Figure 3A,B). Conversely, genes differentially up-expressed in the normal control group were involved in extracellular matrix (ECM) receptor interaction, cyclic adenosine monophosphate (cAMP) signaling pathway, and the positive regulation of stem cell proliferation (Figure 3C).

Transcriptomic comparison between the LPS- and Mito-NP-treated groups revealed that collagen catabolic processes and negative regulation of the response to ROS were enhanced in the LPS group (Figure 3D,E). In contrast, Mito-NPs treatment upregulated pathways related to cell cycle and cAMP signaling (Figure 3F). These results suggest that LPS treatment impairs TSC function, leading to increased apoptosis and matrix degradation, while Mito-NPs may help restore TSCs’ function by modulating gene expression.

### 3.4. Mito-NPs Improve the Functional Phenotype of TSCs

**Figure 4:**
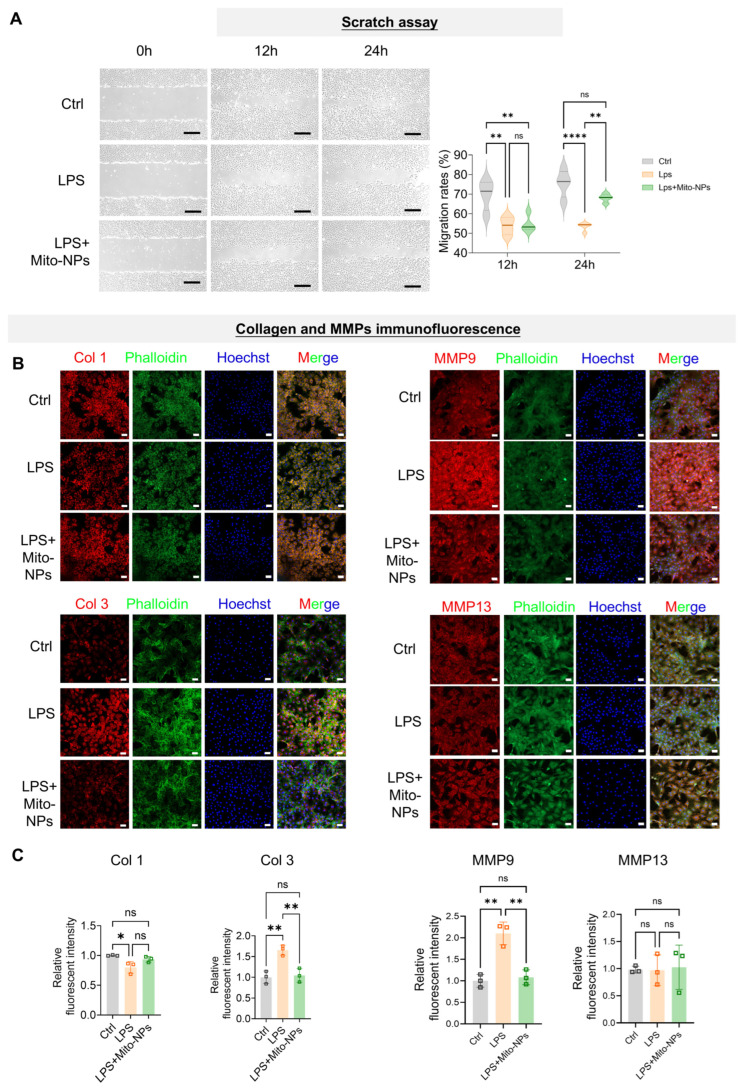
The effect of Mito-NPs on the functional phenotype of TSCs under inflammatory conditions in vitro. (**A**). Representative microscopic images showing the migration rate in TSCs under control, LPS, and LPS + Mito-NPs conditions at 0 h, 12 h, and 24 h and quantification analysis. Scale bar = 200 μm. *n* = 4. (**B**). Representative confocal images showing Col1, Col3, MMP9 and MMP13 intensity in TSC under control, LPS, and LPS + Mito-NPs conditions. Phalloidin labels the filamentous actin (F-actin) and DAPI labels the nucleus. Scale bar = 50 μm. (**C**). The quantification analysis of Col1, Col3, MMP9 and MMP13. *n* = 3. * *p* < 0.05, ** *p* < 0.01, *** *p* < 0.001, **** *p* < 0.0001; All data are shown as the mean ± SD; Statistical significance was determined by one-way ANOVA with Turkey’s test.

**Figure 5:**
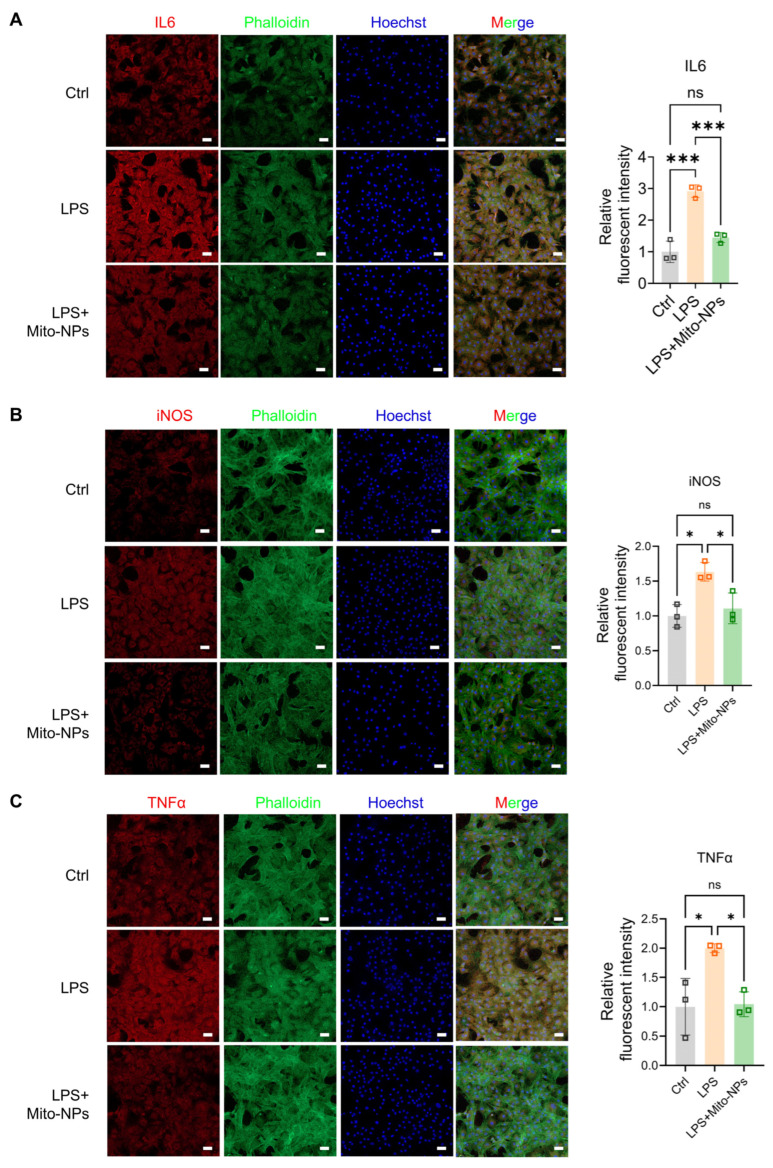
Anti-inflammatory effect of Mito-NPs on TSCs. (**A**). Representative confocal images showing IL6 intensity in TSC under control, LPS, and LPS + Mito-NPs conditions and quantification analysis. (**B**). Representative confocal images showing iNOS intensity in TSC under control, LPS, and LPS + Mito-NPs conditions and quantification analysis. (**C**). Representative confocal images showing TNFα intensity in TSC under control, LPS, and LPS + Mito-NPs conditions and quantification analysis. Phalloidin labels the filamentous actin (F-actin) and DAPI labels the nucleus. Scale bar = 50 μm; *n* = 3. * *p* < 0.05, ** *p* < 0.01, *** *p* < 0.001; All data are shown as the mean ± SD; Statistical significance was determined by one-way ANOVA with Turkey’s test.

We next evaluated the effect of Mito-NP delivery on functional phenotypic changes in primary TSCs. Immunocytochemical staining demonstrated that LPS exposure significantly reduced Ki67 expression, which indicates suppressed cell proliferation. Conversely, treatment with Mito-NPs markedly increased Ki67 expression, suggesting an enhanced capacity for cell proliferation (Supplementary Figure S4). The cell scratch assay demonstrated that LPS treatment significantly inhibited TSC migration, with average healing rates of 50% at 12 h (compared to 72% in the control group) and 53% at 24 h (Figure 4A). Mito-NPs treatment significantly restored TSC migration, increasing the scratch healing rate to 69% at 24 h (control group: 75%) (Figure 4A). Immunofluorescence staining revealed that LPS-induced inflammation significantly decreased Col 1 expression but increased Col 3 and MMP9 levels compared to the control (Figure 4B,C). Mito-NPs treatment reversed this pattern, restoring the expression of all three proteins to near-physiological levels (Figure 4B,C). In addition, LPS treatment elevated the fluorescence intensity of inflammatory factors IL-6, TNF-α, and iNOS (Figure 5A–C). Following Mito-NPs treatment, Col 1 expression increased, Col 3 and MMP9 expression decreased, and the fluorescence intensity of IL-6, TNF-α, and iNOS decreased (Figure 4B and Figure 5A–C). These findings indicate that Mito-NPs effectively improve the inflammatory phenotype of TSCs and promote functional collagen synthesis.

### 3.5. Mito-NPs Improve Motor Function After Tendon Injury in Rats

Mito-NPs were encapsulated in a ROS-responsive TSPBA-PVA hydrogel, referred to as Mito-NPs@HG, using a previously reported maturation method [27]. The hydrogel’s responsiveness to 100 μM H_2_O_2_ was confirmed in vitro by a 40% reduction in wet weight after 4 h of treatment (Supplementary Figure S5A,B). In the PBS buffer, the hydrogel underwent sustained degradation over 72 h, with a concomitant steady release of Mito-NPs (Supplementary Figure S5C,D). Local implantation of Mito-NPs@HG into the injured rat. Achilles tendon enabled targeted delivery and responsive release of Mito-NPs in the inflammatory microenvironment.

**Figure 6:**
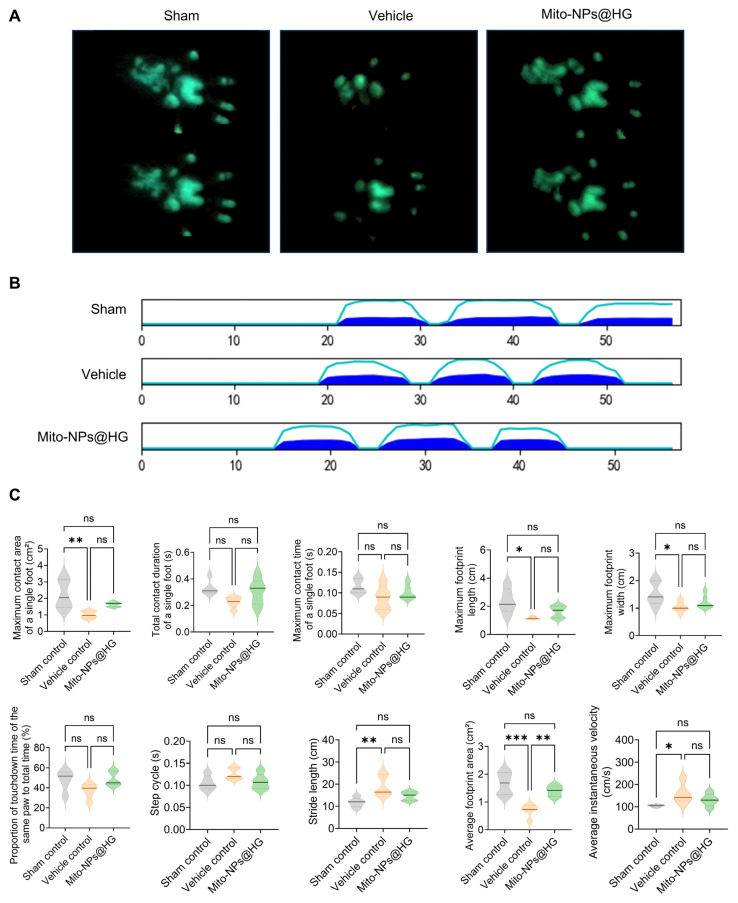
Gait analysis of rats. (**A**). Representative footprint intensity images of the Sham, Vehicle, and Mito-NPs@HG at 4 weeks after treatment. (**B**). Representative image showing landing pressure from the three groups. Line, Maximum intemsity. Color, Mean intensity of the corresponding print. (**C**). The quantification analysis of key gait parameters. *n* = 5. * *p* < 0.05, ** *p* < 0.01, *** *p* < 0.001; All data are shown as the mean ± SD; Statistical significance was determined by one-way ANOVA with Turkey’s test.

At four weeks post-operation, functional recovery was assessed via gait analysis. The paw prints of rats in the Vehicle control group exhibited diminished fluorescent intensity and a smaller contact area compared to the Sham group. In contrast, the Mito-NPs@HG groups displayed a restoration of these parameters, with a more intense and widespread contact pattern (Figure 6A). Quantitative analysis revealed that rats in the Vehicle control group exhibited reduced landing pressure, maximum contact area of a single foot, maximum contact length, maximum contact width, and average footprint area compared to the Sham control group (Figure 6A–C). In contrast, the Mito-NPs@HG implant group demonstrated increased average footprint area and improved motor function, approaching the levels observed in the sham control group. These results demonstrated that Mito-NPs@HG treatment facilitated a recovery towards normal gait function.

### 3.6. Mito-NPs Promote Histological Repair After Tendon Injury

**Figure 7:**
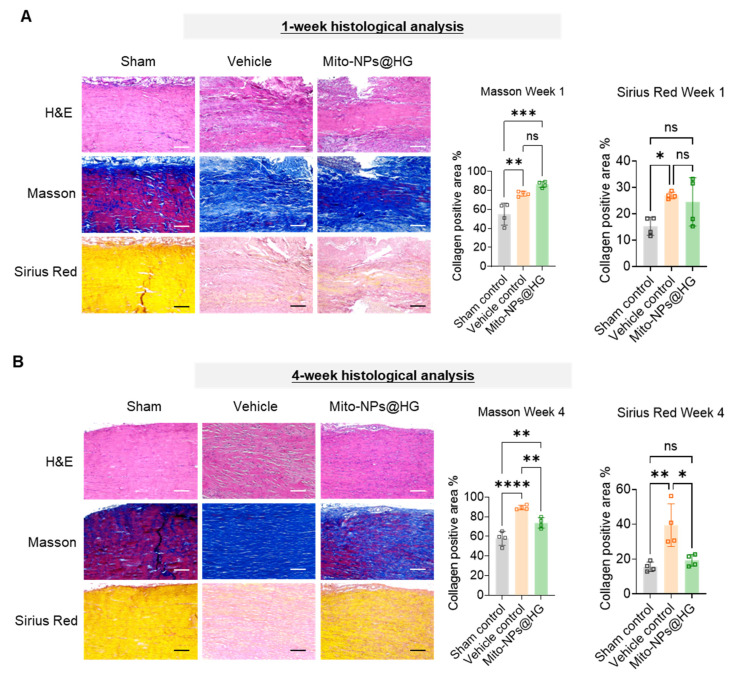
Therapeutic effects of Mito-NPs in vivo. (**A**). Representative images of H&E staining (upper panel), Masson’s trichrome staining (middle panel), and Sirius red staining (lower panel) of tendons of rats from three different groups at 1 week postoperatively and the quantification of collagen positive area. Scale bar indicates 100 µm. (**B**). Representative images of H&E staining (upper panel), Masson’s trichrome staining (middle panel), and Sirius red staining (lower panel) of tendons of rats from three different groups at 4 weeks postoperatively and the quantification of collagen positive area. Scale bar indicates 100 µm *n* = 4. * *p* < 0.05, ** *p* < 0.01, *** *p* < 0.001, **** *p* < 0.0001; All data are shown as the mean ± SD; Statistical significance was determined by one-way ANOVA with Turkey’s test.

Histological evaluation of rat Achilles tendon injury sites was performed at 1 and 4 weeks post-operation. At 1 week, the Vehicle control group exhibited extensive inflammatory cell infiltration, disorganized tissue morphology, and loosely arranged collagen fibers (Figure 7A). In contrast, the Mito-NPs@HG group displayed a more regular tissue structure. At 4 weeks, the Vehicle control group still showed prominent scar tissue and disordered collagen fiber bundles, whereas the Mito-NPs@HG group had significantly reduced scar tissue and closely aligned, parallel collagen fibers (Figure 7B). Masson and Sirius Red staining revealed that the proportion of collagen-positive area in the Vehicle control group was higher than that in the Sham control group at both time points, indicating ongoing collagen remodeling (Figure 7A,B). Although the collagen-positive area increased at 1 week in the Mito-NPs@HG group, it decreased by 4 weeks, ultimately reaching a distribution closer to that of normal Achilles tendon tissue.

**Figure 8:**
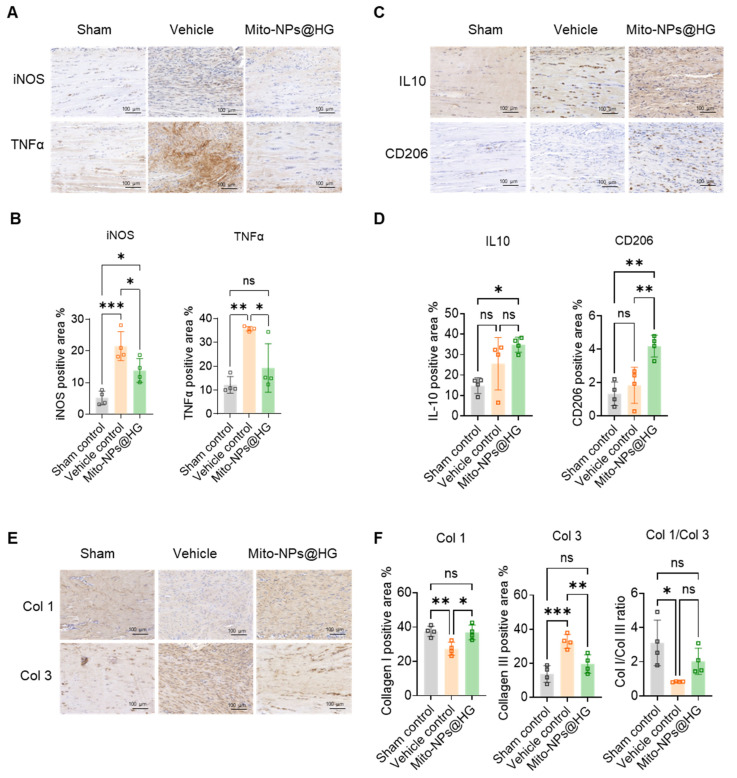
Immunohistochemical analysis of rat tendons. (**A**). Representative images of immunofluorescent staining of iNOS (upper panel) and TNFα (lower panel) of sections of each group at 1 week postoperatively. Scale bar = 100 µm. (**B**). Quantification of iNOS (left panel) and TNFα (right panel) expression in different groups. (**C**). Representative images of immunofluorescent staining of IL10 (upper panel) and CD206 (lower panel) of sections of each group at 1 week postoperatively. Scale bar = 100 µm. (**D**). Quantification of IL10 (left panel) and CD206 (right panel) expression in different groups. (**E**). Representative images of immunofluorescent staining of Col 1 (upper panel) and Col 3 (lower panel) of sections of each group at 4 weeks postoperatively. Scale bar = 100 µm. (**F**). Quantification analysis of Col 1 (left panel), Col 3 (middle panel) and Col 1/Col 3 ratio (right panel) in different groups. *n* = 4. * *p* < 0.05, ** *p* < 0.01, *** *p* < 0.001; All data are shown as the mean ± SD; Statistical significance was determined by one-way ANOVA with Turkey’s test.

Immunohistochemistry results indicated that at 1-week post-operation, the Vehicle control group had a higher proportion of inducible iNOS and TNF-α positive staining than the Sham control group (Figure 8A,B). In the Mito-NPs@HG group, expression of these inflammatory factors decreased, while anti-inflammatory IL-10 and CD206 were significantly up-regulated (Figure 8C,D). At 4 weeks, the Vehicle control group showed increased Col 3, decreased Col 1, and reduced Col 1/Col 3 ratio compared with the Sham control group (Figure 8E,F). Conversely, the Mito-NPs@HG group showed a significant increase in the Col 1/Col 3 ratio (Figure 8E,F), promoting a collagen composition more typical of functional tendon tissue.

### 3.7. In Vivo Transcriptional Pattern Post Mito-NPs Delivery

**Figure 9:**
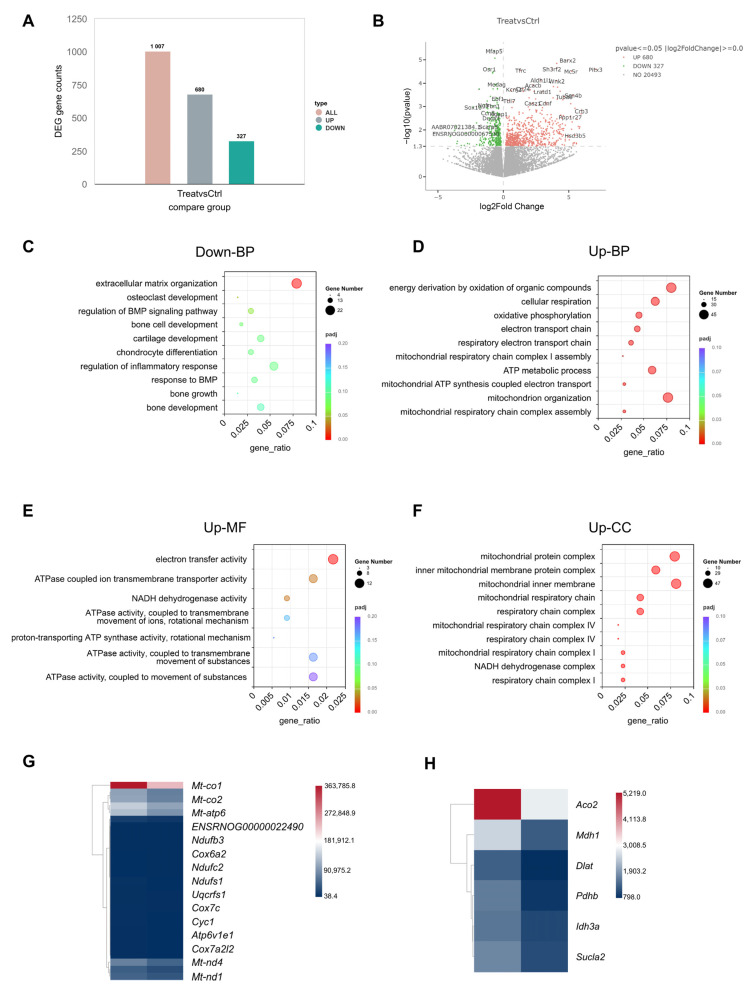
Transcriptome analysis of rat tendons treated with Mito-NPs@HG. (**A**,**B**). Histogram (**A**) and volcano plot (**B**) showing the differentially expressed genes between control and treatment (Mito-NPs@HG) groups at 4 weeks postoperatively. (**C**–**F**) Enrichment analysis was conducted on down-regulated genes in the Mito-NPs@HG group. (**D**–**F**) Gene Ontology (GO) enrichment analysis on up-regulated and down-regulated gene sets in the Mito-NPs@HG group. BP, biological process; MF, molecular function; CC, cellular component. (**G**,**H**). Heatmap analysis was conducted on key genes related to (**G**) mitochondrial oxidative phosphorylation and (**H**) tricarboxylic acid (TCA) cycle in the two groups.

At 4 weeks post-operation, Achilles tendon tissue was collected for transcriptome sequencing to analyze changes in mitochondrial metabolism-related gene expression. Approximately 1007 genes were differentially expressed between the Mito-NPs@HG and Vehicle control groups, with 680 up-regulated and 327 down-regulated in the Mito-NPs@HG group (Figure 9A,B). Enrichment analysis indicated that down-regulated genes in the Mito-NPs@HG group were primarily associated with bone and cartilage remodeling pathways, including osteoclast and bone cell development (Figure 9C). Up-regulated genes were mainly involved in mitochondrial respiration, OXPHOS, and the electron transport chain (Figure 9D–F). Further analysis revealed significant up-regulation of key genes related to mitochondrial OXPHOS and the tricarboxylic acid (TCA) cycle in the Mito-NPs@HG group (Figure 9G,H). These findings confirm that Mito-NPs@HG effectively improves mitochondrial metabolic function in injured Achilles tendon tissue.

## 4. Discussion

Tendon tissue regeneration remains a significant clinical challenge. Mitochondrial dysfunction, exacerbated by sustained oxidative stress and inflammation, contributes to ongoing tissue degeneration [14,18]. This study introduces a therapeutic approach that involves the transplantation of Mito-NPs@HG nanoparticles at the site of Achilles tendon injury to facilitate tissue repair. These nanoparticles are engineered to promote mitochondrial metabolism and enhance TSC function. In vivo experiments demonstrate that Mito-NPs@HG accelerates Achilles tendon repair in a rat model, suggesting potential applications for the treatment of additional musculoskeletal injuries.

Achilles tendon rupture frequently occurs in athletes and is primarily managed through surgical interventions, including direct suture and autologous transplantation [33]. Direct suture often results in complications such as tendon re-tear and peritendinous adhesion, which limit joint mobility [34]. Autologous transplantation may cause injury at the donor site, leading to pain or functional impairment [35]. Non-surgical treatments avoid surgical trauma but are associated with prolonged healing times and a higher likelihood of inadequate tendon healing, which negatively impacts patients’ motor function and daily quality of life [36,37]. Biological therapies represent a promising alternative for Achilles tendon repair [38]. This study specifically targets mitochondrial dysfunction, aiming to restore the metabolic activity of TSCs by directly supplementing functional mitochondria and modulating the inflammatory microenvironment.

Mitochondrial delivery represents an emerging approach for tissue repair [39,40]. Initial strategies involved transplantation isolated mitochondria directly into injury sites to supplement functional mitochondria [41,42]. However, these mitochondria may be rapidly cleared by the immune system, exhibit short half-lives, and lack cellular targeting, resulting in unstable therapeutic efficacy. Unlike endocytosis-dependent delivery, liposome-encapsulated mitochondria may directly fuse with target cell membranes, forming channels for direct mitochondrial release into the cytoplasm [43]. Here, mitochondria were coated with adipose-derived stem cell (ADSC) membranes to construct Mito-NPs. We hypothesize that, in addition to encapsulation, ADSC membranes may confer properties that enhance therapeutic efficacy, potentially through the following mechanisms. The ADSC membrane is enriched with cell adhesion and signaling molecules, which enhance mitochondrial recognition and improve cellular uptake [44,45]. The biocompatibility of the ADSC membrane reduces immune recognition and phagocytic clearance, thereby extending mitochondrial survival [44]. Furthermore, the ADSC membrane possesses intrinsic anti-inflammatory and reparative properties, which synergize with mitochondrial function to enhance therapeutic outcomes [44,45]. Mito-NPs demonstrated a uniform particle size distribution and stable surface charge. Following mitochondrial encapsulation by extrusion, mitochondrial membrane potential was significantly enhanced. Although the precise mechanism remains unclear, it is hypothesized that mitochondria with abnormal or impaired membrane potential may undergo swelling, which impedes their passage through the extruder membrane and results in a filtering effect. In comparison to treatment with cell membranes alone, ADSC membrane treatment did not produce a similar pro-proliferative effect, as indicated by CCK-8 assays and Ki67 immunofluorescence staining. These results support the efficacy of Mito-NPs for in vivo delivery and preservation of biological activity.

Encapsulating Mito-NPs within a ROS-responsive hydrogel (Mito-NPs@HG) creates a targeted delivery system that enhances therapeutic efficacy [27,46,47]. The ROS-responsive hydrogel enables local retention and sustained release of Mito-NPs, minimizing their loss through diffusion and extending the duration of therapeutic action. This hydrogel detects elevated ROS levels at injury sites, triggering degradation and controlled release of Mito-NPs, thereby achieving on-demand drug delivery and improving utilization efficiency. Experimental results indicate that the hydrogel exhibits a 40% reduction in wet weight within four hours in the presence of 100 μM H_2_O_2_, demonstrating effective ROS responsiveness.

The healing of tendon rupture involves a complex sequence of biological events, including inflammation, proliferation, and remodeling [48]. After injury, local production of inflammatory factors causes acute oxidative stress, leading to cell death, DNA damage, and collagen breakdown in TSCs [49,50]. ROS also leads to M1 macrophage polarization and the release of pro-inflammatory cytokines such as IL-6 and TNF-α, which worsen inflammation and create a cycle of excessive ROS, mitochondrial dysfunction, and increased inflammation [13,51,52]. Mitochondrial dysfunction further disrupts energy metabolism and decreases ATP production in TSCs, hindering cell growth, movement, and collagen formation [18]. Pharmacological interventions targeting cellular oxidative stress can stabilize mitochondrial membrane potential and ATP synthesis in TSCs, promote the structural regeneration of collagen fibers, and thereby restore mechanical properties and motor function [53]. In this study, Mito-NPs restored energy production and substrate use in TSCs, lowered ROS levels, and broke the cycle of oxidative stress by directly supplying healthy mitochondria. Furthermore, mitochondrial delivery modulated inflammatory factors, reduced the release of pro-inflammatory mediators (IL-6, TNF-α, iNOS), increased anti-inflammatory cytokine (IL-10) expression, and supported the remodeling of the inflammatory microenvironment.

Col 1 molecules, characterized by a triple-helical structure, assemble into fibrils, which further form fibers and bundles, ultimately constructing the tendon unit [54]. In the early stage of Achilles tendon injury (1 week), despite an overall increase in collagen proportion (as indicated by Masson staining), Col 1 expression decreases while Col 3 expression increases with escalating inflammation. This observation is consistent with collagen alterations reported in clinical studies following tendon injury [55,56,57]. Notably, Mito-NPs promoted the early production of Col 1.

In preclinical tendon repair, ADSC membrane-coated mitochondrial transfer (Mito-NPs@HG) represents an alternative to cell-based therapies and may provide more targeted regulation of energy metabolism. In contrast to exosome-based approaches, it avoids complex purification steps and directly delivers mitochondria. Its ROS-responsive hydrogel allows for controlled, localized release, overcoming the limited tissue retention of small molecules. By directly addressing mitochondrial dysfunction, the primary cause of TSC impairment, rather than only alleviating symptoms, this approach improves therapeutic efficiency and streamlines application. Consequently, Mito-NPs@HG emerges as a promising candidate for tendon injury treatment.

## 5. Conclusions

Our study demonstrates that Mito-NPs restore TSC mitochondrial metabolism, modulate gene expression, and improve functional phenotype under inflammatory conditions. Encapsulating Mito-NPs in a ROS-responsive hydrogel enhances their in vivo therapeutic efficacy, promoting histological repair and functional recovery in a rat Achilles tendon injury model. This work provides a novel, biocompatible mitochondrial delivery system for tendon repair, with potential applications in other musculoskeletal injuries characterized by mitochondrial dysfunction.

## Data Availability

The original contributions presented in this study are included in the article/Supplementary Materials. Further inquiries can be directed to the corresponding authors.

## Supplementary Materials

The following supporting information can be downloaded at: https://www.mdpi.com/article/10.3390/jfb17030119/s1.
