# Supplementary material for: Adipose-Derived Stem Cell Membrane-Coated Mitochondria Restore Tendon Stromal Cell Function Through Metabolic Reprogramming and Promote Achilles Tendon Healing
Source: J Funct Biomater. 2026 Mar 2;17(3):119. doi: 10.3390/jfb17030119 (PMC13028381; doi:10.3390/jfb17030119)
Supplement: Supplementary file 1 [file jfb-17-00119-s001.zip › jfb-3988073-supplementary.pdf]

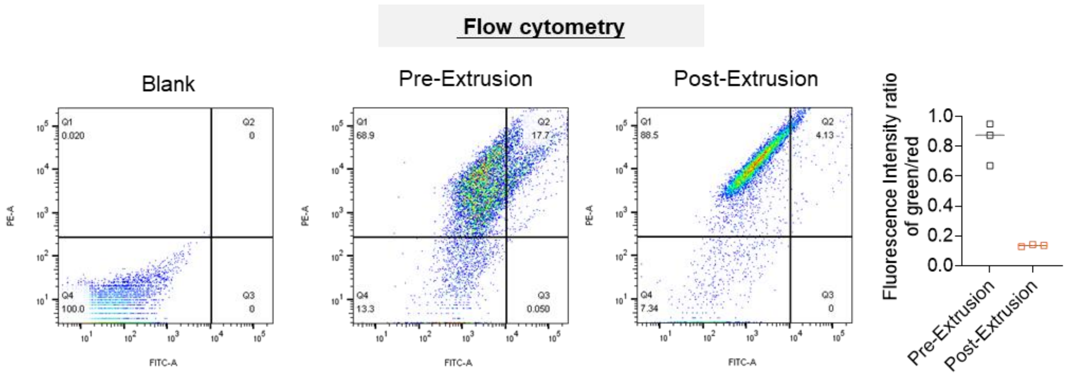

Supplementary Figure S1. Flow cytometry analysis of changes in mitochondrial membrane potential before and after extrusion following JC-1 staining. A decreased ratio of green fluorescence (FITC) to red fluorescence (PE) indicates an increase in mitochondrial membrane potential. Blank: Unstained mitochondria before extrusion. Pre-Extrusion: Mitochondria before extrusion. Post-Extrusion: Mitochondria after extrusion

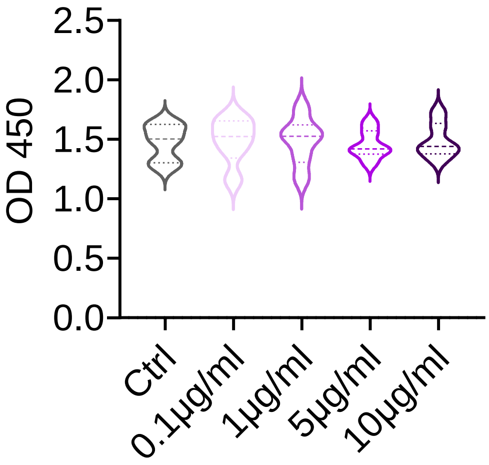

Supplementary Figure S2. CCK-8 assay results showing the effects of ADSC membrane at different doses on the proliferation of TSCs.

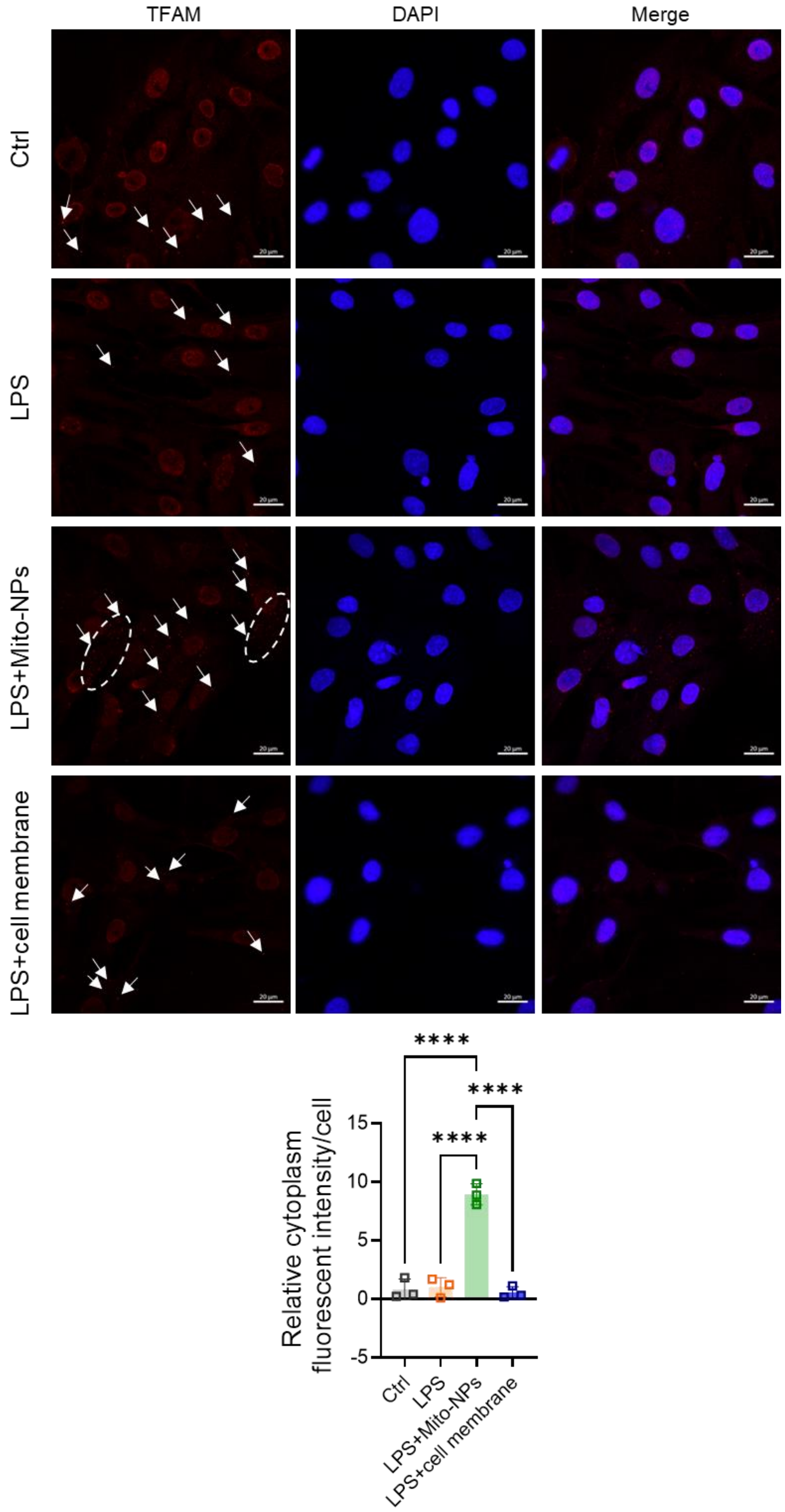

TFAM
DAPI
Merge
Ctrl
LPS
LPS+Mito-NPs
LPS+cell membrane
20 μm
****
****
****
Relative cytoplasm fluorescent intensity/cell
15
10
5
0
-5
Ctrl
LPS
LPS+Mito-NPs
LPS+cell membrane

Supplementary Figure S3. Representative confocal immunofluorescence images of TFAM in TSCs under control, LPS, LPS + Mito-NPs, and LPS + ADSC membrane conditions, along with the corresponding quantitative analysis. Nuclei were counterstained with DAPI (blue fluorescence). Red: TFAM. Arrows and dashed lines indicate the positive staining areas. Scale bar = 20 μm. *p < 0.05, **p < 0.01, ***p < 0.001; All data are shown as the mean ± SD; Statistical significance was determined by one-way ANOVA with Turkey's test.

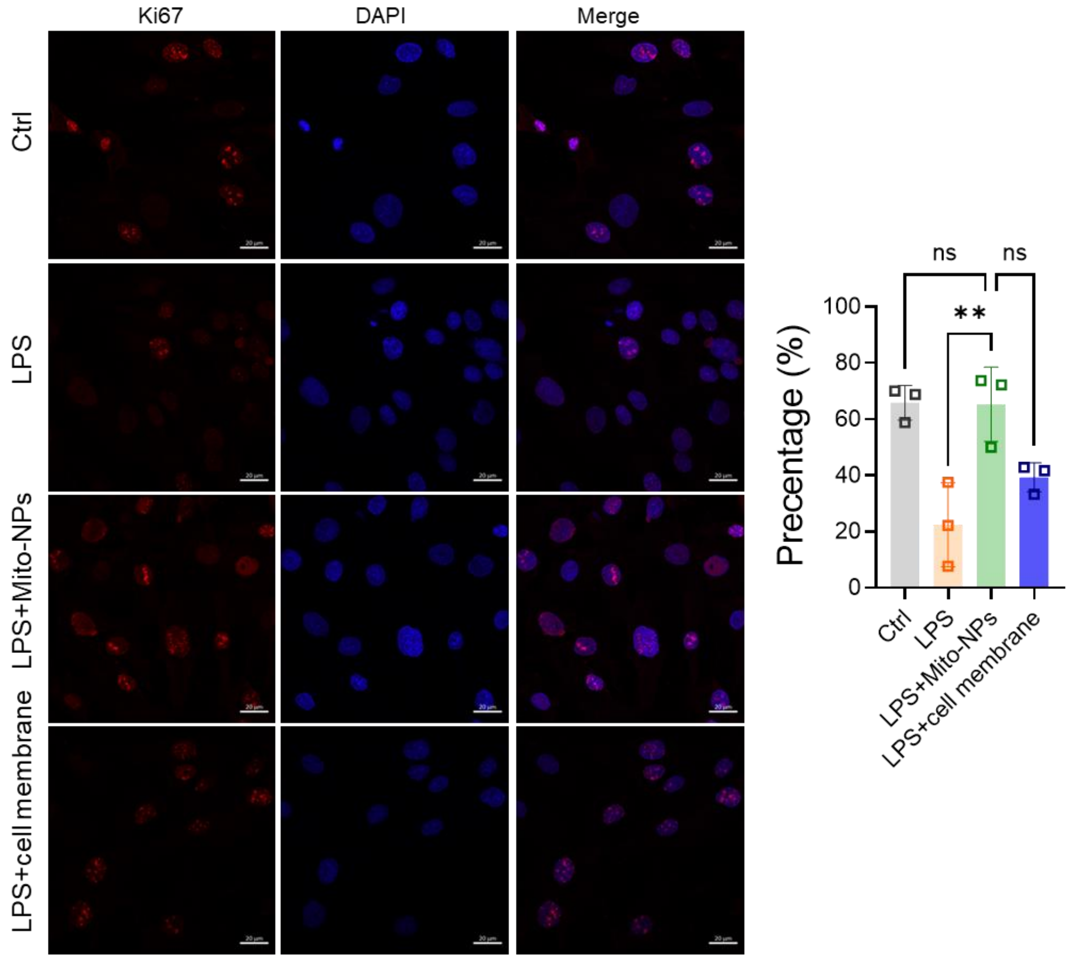

Supplementary Figure S4. Representative confocal immunofluorescence images of Ki67 in TSCs under control, LPS, LPS + Mito-NPs, and LPS + ADSC membrane conditions, along with the corresponding quantitative analysis. Nuclei were counterstained with DAPI (blue fluorescence). Red: Ki67. Arrows and dashed lines indicate the positive staining areas. Scale bar = 20 μm. *p < 0.05, **p < 0.01, ***p < 0.001; All data are shown as the mean ± SD; Statistical significance was determined by one-way ANOVA with Turkey's test.

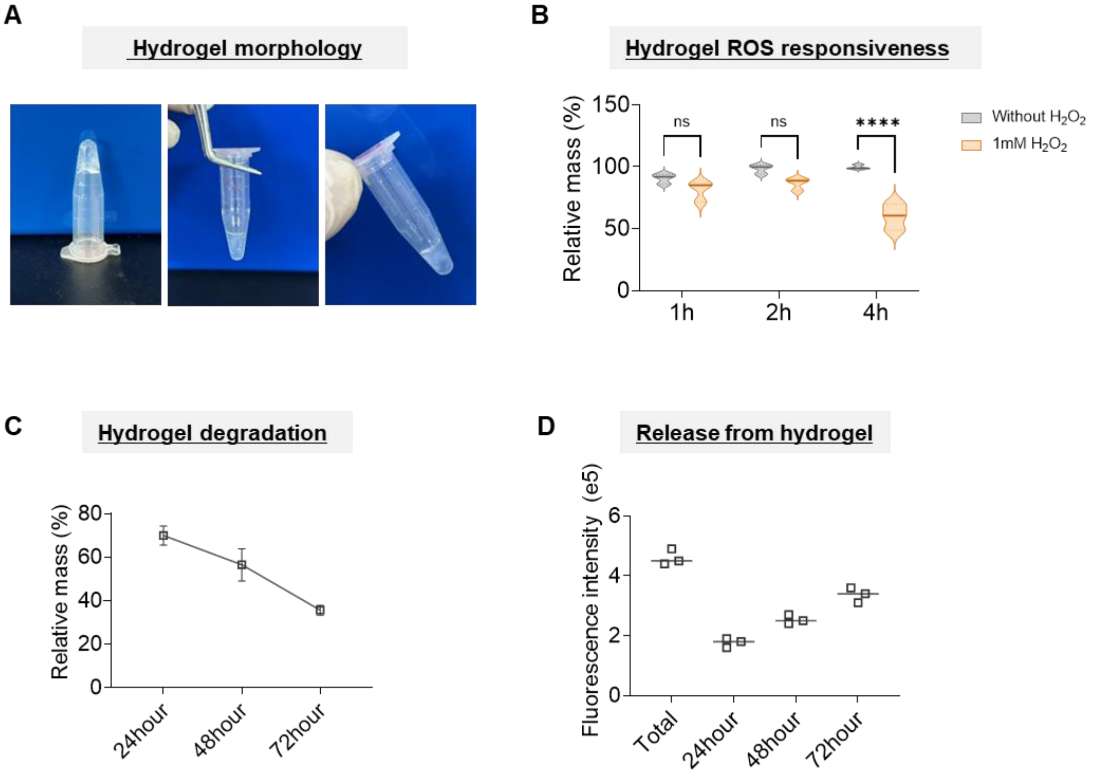

Supplementary Figure S5. Characterization of hydrogel. A. The morphology of hydrogel. B. The ROS responsiveness of hydrogel. C. Degradation profile of the hydrogel within 72 hours evaluated by the wet weight method. D. Cumulative release kinetics of Mito-NPs from the hydrogel over a 72 hour period. $*p < 0.05$, $**p < 0.01$, $***p < 0.001$; All data are shown as the mean ± SD) Statistical significance was determined by two-way ANOVA with Fisher's LSD test (B).
